# Bio-electrical impedance analysis for perioperative fluid evaluation in open major abdominal surgery

**DOI:** 10.1007/s10877-019-00334-8

**Published:** 2019-06-14

**Authors:** Adi-Ionut Ciumanghel, Ioana Grigoras, Dimitrie Siriopol, Mihaela Blaj, Daniel-Mihai Rusu, Gabriela Raluca Grigorasi, Alexandru Razvan Igna, Oana Duca, Ianis Siriopol, Adrian Covic

**Affiliations:** 1 ‟Grigore T. Popa” University of Medicine and Pharmacy, Iasi, Romania; 2 Anesthesia and Intensive Care Department, ‟Sf. Spiridon” University Hospital, Iasi, Romania; 3 Anesthesia and Intensive Care Department, Regional Institute of Oncology, Iasi, Romania; 4 Nephrology Department, ‟Dr. C.I. Parhon” University Hospital, Iasi, Romania

**Keywords:** Bioimpedance, Major abdominal surgery, Fluid balance, Postoperative organ dysfunction, Outcome

## Abstract

Water retention and intercompartmental redistribution occur frequently in association with adverse postoperative outcomes, yet the available strategies for non-invasive assessment are limited. One such approach for evaluating body water composition in various circumstances is bio-electrical impedance analysis (BIA). This study aims to appraise the usefulness of the Body Composition Monitor (BCM, Fresenius Medical Care, Germany) in assessing body fluid composition and intercompartmental shifts before and after open major abdominal surgery. This prospective, clinician blinded observational study enrolled all the patients scheduled consecutively for elective major open abdominal surgery during a 1-year period starting from January 1st, 2016. BIA parameters—total body water (TBW), extracellular water (ECW), intracellular water (ICW), absolute fluid overload (AFO), and relative fluid overload (RFO) were measured before and after surgery. The results were compared with fluid balance and outcome parameters such as organ dysfunction, ICU-and hospital length of stay (-LOS). The study population included 71 patients aged 60.2 ± 12 of whom 60.6% men and with a BMI of 26.3 ± 5.1 kg/m^2^. Postoperative acute kidney injury, respiratory dysfunction, and infections occurred in 14.0%, 19.7% and 28.1% of cases, respectively. The median LOS in ICU was 20 h and the hospital-LOS was 10 days. Positive intraoperative fluid balance (2.4 ± 1.0 L) resulted in a significant increase of TBW (1.4 ± 2.4 L) and of ECW (1.4 ± 1.2 L). Intraoperative fluid balance significantly correlated with TBW change (r = 0.23, p = 0.04) and with AFO change (r = 0.31, p < 0.01). A significant correlation was found between pre- and postoperative AFO and RFO on one hand, and ICU-LOS on the other. BIA may be a useful tool for the perioperative assessment of volume status.

## Introduction

While intraoperative fluid administration is common with patients undergoing surgery, views differ with regard to the type, timing, and volume of the actual intraoperative fluid therapy [1–3]. Intravenous fluids should be prescribed only when explicitly recommended for absolute/relative hypovolemia correction (resuscitation fluids), deficit compensation (replacement fluids) or/and basal requirement provision of water and electrolytes (maintenance fluids) [4].

Traditionally, significant amounts of fluid would be infused during the perioperative period [5, 6] before multiple studies demonstrated that, after major gastrointestinal surgery, excessive intravascular volume may increase postoperative morbidity and mortality while judicious perioperative fluid therapy may improve outcomes [3, 5–8]. Perioperative fluid management based on a goal-directed therapy (GDT) protocol in which specific hemodynamic targets guide fluid and vasoactive drug administration has the potential to lessen postoperative complications [9, 10]. Such an approach may nonetheless also result in infusing larger volumes compared to standard care [9]. An increasing body of data has shown that a more conservative approach to fluid management is beneficial during the perioperative period [11] and during critical illness [12]. However, a recent large trial (RELIEF) failed to demonstrate that restrictive fluid management in major abdominal surgery is better than the more liberal counterpart [13]. The RELIEF study results should be cautiously interpreted and applied due to its several limitations [14]. During critical illness, the ROSE concept of the four fluid phases (resuscitation, optimization, stabilization, and evacuation) may help inform and optimize fluid therapy [4].

Avoiding too much or too little intervention in the perioperative period is a long-recognized goal, but guidance is currently vague: exercise caution with regard to dosage, type, rate, duration, and timing of fluid administration.

The most common early occurrence following major abdominal surgery is fluid overload [15]. Water excess may impair the proper functioning of the lungs [5, 16, 17], heart [5], renal and gastrointestinal system [8, 18]. It may also decrease tissue oxygenation and undermine wound healing by increasing the risk of infection or anastomotic leakage. Moreover, it may prolong the length of stay in ICU [8]. After initial stabilization, conservative goal-directed fluid management focused on negative fluid balance may improve clinical outcomes [4, 19–21]. There are protocols for intravenous fluid therapy [22], but their uniform application implies giving the same volume to different patients and in different circumstances. To address the uniqueness of each patient and situation, rather than infusing a predefined amount of fluid, the goal should be to tailor fluid infusion based on a needs analysis [23, 24].

Regarding the best method for assessing volume status, there is still ongoing debate. Clinical signs are a poor and late indicator of fluid volume and intercompartmental distribution. For intravascular space evaluation, there are several recommended strategies using non-invasive, minimally invasive or invasive monitoring, but few that address extravascular water. Bio-electrical impedance analysis (BIA) assesses body composition and estimates total and extracellular water volumes based on the tissue’s capacity to conduct electrical impulses. BIA is currently recommended for appraising fluid status in hemodialysis patients. Some data regarding BIA use in critically ill patients [25], sepsis [26, 27], burn patients [28], and during pregnancy [29] are available, but BIA for perioperative assessment of fluid status and compartment redistribution has not yet been sufficiently researched.

In this study, we evaluate the usefulness of the Body Composition Monitor device (BCM, Fresenius Medical Care, Germany) for the assessment of body fluid composition and intercompartmental shifts before and after open major abdominal surgery. We compare BIA results with intraoperative fluid balance and with outcome parameters: postoperative organ dysfunction, ICU- and hospital length of stay (LOS).

## Materials and methods

### Study design

We conducted a pilot prospective, clinician blinded, observational study at the “Sf. Spiridon” Emergency University Hospital Iasi, Romania for a 1-year period starting January 1st, 2016.

The study was approved by the Research Ethics Committee of the “Sf. Spiridon” Emergency University Hospital Iasi (EC approval number 33750/2015). Before enrolling, all the patients signed an informed consent which included written information about the nature of the study, its aim, the expected advantages and possible risks, and also data protection assurances.

### Patients

All the patients meeting the inclusion criteria were enrolled consecutively during the aforementioned period. The inclusion criteria were: (1) age > 18 years old, (2) open major abdominal surgery, (3) elective surgery. Exclusion criteria were also applied: (1) patient’s refusal, (2) laparoscopic surgery, (3) emergency surgery, (4) conditions that interfered with making accurate BIA measurements: limb amputation, metallic cardiac or joint prostheses, cardiac pacemakers or stents, decompensated cirrhosis.

### Bio-electrical impedance analysis

The principle on which bio-impedance is based is that different tissues have different conductive and resistive properties when a small electric current is applied at different frequencies. The impedance of the body consists of two components: (1) resistance, which is proportional to fluid volume, and (2) reactance, meaning the reciprocal of the capacitance of cell membranes. At a low frequency (5 kHz), impedance indicates extracellular fluid because current does not flow through cell membranes. At frequencies above 100–200 kHz, the current will penetrate the cell membrane and impedance will reflect both extracellular and intracellular fluids [30].

Extracellular and intracellular resistance values are obtained based on the Cole model [31] while TBW, ECW, and ICW are automatically calculated by the BIA device as described by Moissl [32]. Subsequently, extracellular fluid excess can be distinguished from normal body hydration using the Chamney physiological tissue model [33]. This model provides the normal hydration status in a human being of given weight, height and gender, i.e. the expected normal values for ECW and ICW that would usually occur in an individual in normohydration state and with an adequate renal function.

The BIA device also displays absolute fluid overload (AFO), the difference between normal, expected ECW and the actual, measured ECW, expressed in liters, as well as relative fluid overload (RFO), absolute fluid overload/extracellular water ratio (AFO/ECW), expressed in percentages. A negative AFO points to the patient’s underhydration while a positive one indicates overhydration. Based on RFO values, a patient’s hydration status was classified into three categories: dehydrated (RFO < − 10%), normohydrated (− 10% ≤ RFO ≤+ 15%), hyperhydrated (RFO > 15%).

In our research, BIA was assessed using the Body Composition Monitor (BCM, Fresenius Medical Care, Germany) according to the manufacturer’s recommended technique. First, the patient was placed in supine position on the hospital bed without touching metal objects, with the limbs in slight abduction and palms placed flat on the bed surface. Then, after the skin was cleaned with alcohol, 4 non-recyclable electrodes were attached: 2 on one hand (on the wrist’s bony protuberance and just behind the metacarpals) and 2 on the ipsilateral foot (on the ankle midline, between the medial and lateral malleoli and just behind the metatarsals). Measurements were performed by one of our two trained physicians.

### Study protocol

BIA was performed preoperatively within 1 h before surgery. Data regarding the patient’s body weight and height, gender and age were entered into the BIA device.

Anesthesia was managed according to hospital protocol. All patients were premedicated with 10 mg of diazepam and they fasted overnight for 6–8 h. In the operating room, all patients were monitored before/during anaesthesia using three lead electrocardiography, non-invasive blood pressure measured every 3–5 min, peripheral pulse oximetry, central temperature and end-tidal carbon dioxide concentration. Some of the patients required additional invasive haemodynamic monitoring (Infinity^®^ Delta Draeger multiparameter monitor). General anaesthesia was induced with propofol 1–2 mg/kg, fentanyl 2–3 µg/kg, and atracurium 0.5 mg/kg. Airways were secured with tracheal intubation and anaesthesia was maintained with 2% sevoflurane in 50% air/oxygen mixture, fentanyl, and atracurium intermittent boluses according to clinical judgement. All patients had volume-controlled ventilation with a tidal volume of 6–8 mL/kg and PEEP 5 cm H_2_O. All patients had a urinary catheter inserted after induction and their urinary outputs were accurately monitored.

The decisions regarding the volume and timing of intraoperative fluid administration were made by the anaesthesiologist, who was unaware of BIA measurements in order for the results to reflect current clinical practice. The clinician could choose between crystalloids (0.9% sodium chloride, Ringer’s, and Ringer’s lactate solution) and colloids (hydroxyethyl starch 130/0.4 Voluven^®^, gelatins Gelofusin^®^). In case of a blood loss of more than 500 mL, the hospital protocol for intraoperative fluid administration recommends colloids to be 1/3 of the given volume.

The second BIA assessment was performed within the first postoperative hour in the Post-Anesthesia Care Unit or the Intensive Care Unit.

Fluid management in the postoperative period was recommended by the attending physician (the anaesthesiologist or the surgeon) who acted according to routine clinical practice and was equally unaware of BIA measurements.

### Data collection

The following baseline data were recorded for all patients: gender, age, anthropometric data (height, weight, body mass index—BMI), patient diagnosis and surgical intervention, American Society of Anesthesiologists (ASA) risk classification, co-morbidities (cardiac, respiratory, neurological, renal, liver), preoperative and intraoperative hemodynamic parameters (heart rate, systolic/diastolic blood pressure), intraoperative blood loss, urine output, and hemodynamic support (inotropic and/or vasoactive drugs). Intraoperative blood loss was assessed by measuring blood in suction bottles, blood-soaked mops and gauze pieces. Information regarding the volume and type of fluid administered during surgery and in the first two postoperative days was also collected.

The intraoperative fluid balance was calculated by subtracting the losses (blood loss + urinary output, without considering insensible losses) from the infused volumes (crystalloids ± colloids ± blood transfusion). In the postoperative period, we recorded the fluid balance on the 1st and 2nd days. The daily fluid balance was calculated from the patient’s medical records as the difference between fluid intake (intravenous + enteral) and fluid output (urine volume + tube drainages, not including insensible losses). The cumulative fluid balance (CFB) was also calculated as the sum total of the fluid balances recorded intraoperatively as well as on the 1st and 2nd postoperative days. The cumulative fluid overload (CFO) was calculated by dividing the CFB by the preoperative weight of each patient, and the result was expressed as a percentage. With regard to CFO, we divided patients into two groups: normally hydrated (NH) in case of CFO < 5% and fluid overloaded (FO) if CFO ≥ 5%. Standard hematological and biochemical values (white blood cell count, haemoglobin, electrolytes, urea, creatinine, bilirubin, lactate, alkaline reserve, and albumin) were recorded preoperatively and then daily during the postoperative ICU hospitalization period.

All patients were monitored during their hospital stay for timely detection of organ dysfunction or complications. Acute kidney injury was defined according to the KDIGO criteria [34]. Respiratory dysfunction was acknowledged as such when as at least one of the following conditions was present: postoperative mechanical ventilation for ≥ 6 h, arterial blood oxygen partial pressure/inspired oxygen fraction ratio (PaO_2_/FiO_2_) < 300, reintubation or difficult weaning of mechanical ventilation. To diagnose infection, we used the Centers for Disease Control 2008 guidelines [35]. We also recorded ICU- and hospital-LOS, as well as the patient’s status upon discharge (survivor/non-survivor).

### Statistical analysis

Continuous variables are reported as mean ± SD, median and inter-quartile range or as frequency percentages, as appropriate. The Shapiro–Wilk test was conducted to appraise the distribution of continuous variables. Logarithmic transformation was performed for variables with non-parametric distribution. Pre- and postoperative comparisons among patients were made using the paired *t* test. The relationship between variables was assessed using the Pearson correlation coefficient.

All analyses were undertaken using Stata SE software, version 12 (Stata Statistical Software: Release 12. College Station, TX: StataCorp LP.). A two-tailed p < 0.05 was considered significant.

## Results

### Patients

Of the 164 patients with elective major open abdominal surgery performed during the aforementioned period, 71 met all the inclusion criteria and were enrolled in the study.

### Description of study population

Enrolled patients had a mean age of 60.2 ± 12.0 years, a mean BMI of 26.3 ± 5.1 kg/m^2^, and 43 (60.6%) were male. The median duration of anaesthesia was 180 (140–250) min. The majority of patients were ASA 3 (54.9%) and the most frequent type of surgery involved the gastrointestinal tract (Table 1).

**Table 1:** Descriptive statistics

	All (71 pts)	NH group CFO < 5% (49 pts)	FO group CFO ≥ 5% (22 pts)	p*
Age, years	60.2 ± 12.0	59.7 ± 12.8	61.3 ± 10.1	0.574
Male, n (%)	43 (60.6)	28 (57.1)	15 (68.1)	0.382
BMI, kg/m^2^	26.3 ± 5.1	27.4 ± 5.3	23.9 ± 3.8	**0.002**
ASA, n (%)				
I	2 (2.8)	1(2.1)	1(4.5)	
II	30 (42.3)	20 (40.8)	10 (45.4)	
III	39 (54.9)	28 (57.1)	11 (50)	
Preoperative parameters				
Hemoglobin, g/dL	11.9 ± 2.3	12.5 ± 2.2	10.7 ± 2.2	**0.003**
Serum Na, mEq/L	140 ± 3.4	139.4 ± 3.8	140.4 ± 2.0	0.270
Serum K, mEq/L	4.2 ± 0.4	4.2 ± 0.3	4.4 ± 0.5	**0.020**
Serum Cl, mEq/L	104. ± 4.5	103 ± 5.1	105 ± 2.8	0.174
Serum albumin, g/dL	3.6 ± 0.6	3.7 ± 0.6	3.5 ± 0.7	0.185
Lactate, mg/dL	18.5 ± 2.4	18.5 ± 2.4	18.5 ± 2.4	0.941
Serum bicarbonate, mmol/L	25.5 ± 2.9	24.9 ± 2.5	26.9 ± 3.3	**0.008**
Serum creatinine, mg/dL	0.8 ± 0.1	0.8 ± 0.1	0.7 ± 0.1	0.183
Preoperative SBP, mmHg	136 ± 24.1	139 ± 24.0	129 ± 23.6	0.156
Preoperative DBP, mmHg	82 ± 12.8	88 ± 11.9	76 ± 13.2	**0.026**
Type of surgery, n (%)				
*Hemicolectomy*	27 (38.0)	23 (46.9)	4 (18.1)	**0.021**
*Duodenopancreatectomy*	10 (14.1)	2 (4.0)	8 (36.3)	**< 0.001**
*Anterior rectum resection*	7 (9.9)	7 (14.2)	0 (0)	–
*Hysterectomy*	7 (9.9)	6 (12.2)	1 (4.5)	0.317
*Gastrectomy*	6 (8.5)	5 (10.2)	1 (4.5)	0.431
*Esophagectomy*	6 (8.5)	1 (2.0)	5 (22.7)	**0.004**
*Aorto*-*femoral by*-*pass*	5 (7.0)	2 (4.0)	3 (13.6)	0.148
*Total colectomy*	2 (2.8)	1 (2.0)	1 (4.5)	0.558
*Hepatectomy*	1 (1.4)	1 (2.0)	0 (0)	–
Surgery duration, median (range), min	180 (140–250)	170 (70–315)	300 (140–540)	**< 0.001**
Intraoperative parameters				
Total fluids, L	3.7 ± 1.5	3.1 ± 1.1	5.0 ± 1.6	**< 0.001**
Total fluid, mL/kg/h	15.2 ± 5.5	14.2 ± 4.4	17.5 ± 7.1	**0.020**
Crystalloids, L	2.0 ± 0.6	1.8 ± 0.6	2.1 ± 0.6	0.217
Colloids, L	1.3 ± 0.9	0.9 ± 0.6	2.1 ± 0.9	**< 0.001**
Diuresis, L	0.5 ± 0.4	0.3 ± 0.3	0.7 ± 0.6	**0.010**
Blood loss, median (IQR), L	0.6 (0.1–1.1)	0.6 (0.1–1.0)	0.5 (0.0–1.6)	0.490
Intraoperative fluid balance, L	2.4 ± 1.0	2.0 ± 0.8	3.4 ± 0.9	**< 0.001**
Intraoperative fluid balance, mL/kg/h	10.3 ± 5.1	9.5 ± 4.3	12.2 ± 6.6	**0.042**
Mean SBP, mmHg	116.4 ± 18.4	117 ± 11.5	113 ± 9.8	0.216
Mean DBP, mmHg	71.3 ± 10.7	70 ± 10.7	72 ± 11.2	0.321
Postoperative parameters				
1st day hemoglobin, g/dL	11.2 ± 1.3	11.4 ± 1.2	10.7 ± 1.4	0.067
1st day serum albumin, g/dL	3.0 ± 0.7	3.3 ± 0.5	2.5 ± 0.7	**< 0.001**
1st day serum creatinine, mg/dL	0.8 ± 0.2	0.8 ± 0.2	0.8 ± 0.2	0.743
2nd day hemoglobin, g/dL	10.7 ± 1.4	10.9 ± 1.2	10.3 ± 1.5	0.053
2nd day serum albumin, g/dL	3.0 ± 0.6	3.2 ± 0.5	2.5 ± 0.6	**< 0.001**
Day 1 fluid balance, L	0.4 ± 0.9	0.1 ± 0.9	0.9 ± 0.9	**0.002**
Day 2 fluid balance, L	0.1 ± 0.7	− 0.1 ± 0.6	0.5 ± 0.8	**0.023**
Cumulative fluid balance, L	2.9 ± 1.9	2.0 ± 1.4	4.8 ± 1.5	**< 0.001**
Outcomes				
Respiratory dysfunction, n (%)	14 (19.7)	3 (6.1)	11 (50.0)	**< 0.001**
AKI, n (%)	10 (14.0)	5 (10.2)	5 (22.7)	0.163
Infection, n (%)	20 (28.1)	12 (24.4)	8 (36.3)	0.306
ICU stay ≥ 2 days, n (%)	19 (26.7)	6 (10.2)	13 (59)	**< 0.001**
ICU LOS, median (range) h	20 (1–528)	5 (1–144)	75 (4–528)	**< 0.001**
Hospital LOS, median (range) days	10 (4–37)	17 (7–35)	23 (9–60)	0.178
Mortality, n (%)	4 (5.6)	1 (2.0)	3 (13.6)	0.051

Data are expressed as mean ± SD, median with IQR, or percent frequency, as appropriate. Bold values are statistically significant

AKI, acute kidney injury; BMI, body mass index; CFO, cumulative fluid overload; Cl, chloride; ICU, intensive care unit; DBP, diastolic blood pressure; FO group, fluid overload group; ICW, intracellular water; K, potassium; LOS, length of stay; Na, sodium; NH group, normohydration group, SBP, systolic blood pressure

* Comparison between groups

### Fluid balance

The mean intraoperative fluid volume (crystalloids and colloids) was 3.7 ± 1.5 L, equivalent to 15.2 ± 5.5 mL/kg/h of surgery. The median blood loss was 0.6 (0.1–1.1) L and the mean urinary output was 0.5 ± 0.4 L, resulting in a positive mean intraoperative fluid balance of 2.4 ± 1.0 L. The postoperative fluid balance was positive on both days following the intervention, but more so on the 1st day (Table 1). The mean cumulative fluid balance was also positive (2.9 ± 1.9 L).

### Outcome parameters

Postoperative AKI occurred in 10 cases (14.0%), respiratory dysfunction ensued in 14 cases (19.7%), and developed 20 patients (28.1%) developed infections. The median length of stay in ICU was 20 h (ranging from 1 to 528 h) and hospital LOS was 10 days (4-37). 19 patients (26.7%) had an ICU stay longer than 2 days. Four patients (5.6%) died while in hospital.

### BIA measurements

#### Preoperative BIA

The preoperative BIA parameters were TBW 35.9 ± 7.6 L, ECW 16.3 ± 3.5 L, ICW 19.5 ± 4.4 L, ECW/ICW 0.85 ± 0.11, AFO 0.2 ± 1.4 L and RFO 3 (− 3.5 to 7)%. Before the surgery, ten patients (14.1%) were dehydrated (RFO < − 10%) and two (2.8%) were overhydrated (RFO > 15%). We also calculated the ratios between TBW, ICW, ECW, and the patient’s preoperative body weights, and the mean values were 49% (TBW), 26% (ICW), and 23% (ECW).

#### Postoperative BIA

The postoperative BIA parameters were TBW 37.3 ± 7.6 L, ECW 17.7 ± 3.8 L, ICW 19.4 ± 4.2 L, ECW/ICW 0.93 ± 0.12, AFO 1.3 ± 1.5 L and RFO 8 (2 to 12) %. The postoperative status was dehydration (RFO < − 10%) in just one patient (1.4%) and overhydration (RFO > 15%) in 22 patients (31%).

#### Postoperative versus preoperative BIA

Except for ICW, all other BIA parameters increased significantly during the postoperative period: TBW 1.4 ± 2.4 L (p < 0.001), ECW 1.4 ± 1.2 L (p < 0.001), AFO 1.1 ± 1 L (p < 0.001) and RFO 5 (2 to 10)% (p < 0.001). ICW decreased non-significantly (− 0.1 ± 2.2 L) (p = 0.76) (Table 2).

**Table 2:** Bioimpedance volume status assessment in the pre- and postoperative period

BIA parameters	Preoperative	Postoperative	Differences	p
All patients (71 pts)				
TBW, L	35.9 ± 7.6	37.3 ± 7.6	1.4 ± 2.4	< 0.001
ICW, L	19.5 ± 4.4	19.4 ± 4.2	− 0.1 ± 2.2	0.760
ECW, L	16.3 ± 3.5	17.7 ± 3.8	1.4 ± 1.2.	< 0.001
ECW/ICW ratio	0.85 ± 0.11	0.93 ± 0.12	0.08 ± 0.09	< 0.001
AFO, L	0.2 ± 1.4	1.3 ± 1.5	1.1 ± 1.0	< 0.001
RFO, median (IQR), %	3 (− 3.5 to 7)	8 (2 to 12)	5 (2 to 10)	< 0.001
NH group CFO < 5% (49 pts)				
TBW, L	36.8 ± 7.9	37.9 ± 7.8	1.0 ± 2.4	0.003
ICW, L	20.1 ± 4.6	19.7 ± 4.1	− 0.3 ± 2.5	0.412
ECW, L	16.7 ± 3.8	18.1 ± 3.9	1.3 ± 1.3	< 0.001
ECW/ICW ratio	0.84 ± 0.1	0.92 ± 0.11	0.08 ± 0.10	< 0.001
AFO, L	− 0.1 ± 1.4	0.9 ± 1.3	1.0 ± 0.9	< 0.001
RFO, median (IQR), %	− 1 (− 5 to 4)	5 (0 to 9)	5 (2 to 10)	< 0.001
FO group CFO ≥ 5% (22 pts)				
TBW, L	33.8 ± 6.4	35.9 ± 7.2	2.0 ± 2.0	< 0.001
ICW, L	18.1 ± 3.9	18.5 ± 4.4	0.4 ± 1.3	0.154
ECW, L	15.7 ± 2.9	17.1 ± 3.3	1.4 ± 0.9	< 0.001
ECW/ICW ratio	0.88	0.94	0.06 ± 0.08	< 0.001
AFO, L	0.9 ± 1.0	2.2 ± 1.4	1.3 ± 1.2	< 0.001
RFO, median (IQR), %	6.5 (4 to 8)	13.5 (7.5 to 17.5)	5.5 (2 to 9.7)	< 0.001

Data are expressed as mean ± SD

*AFO* absolute fluid overload, *CFO* cumulative fluid overload, *ECW* extracellular water, * FO group* fluid overload group, *ICW* intracellular water, *NH group* normohydration group,* RFO* relative fluid overload, *TBW* total body water

#### BIA versus fluid balance

**Fig. 1:**
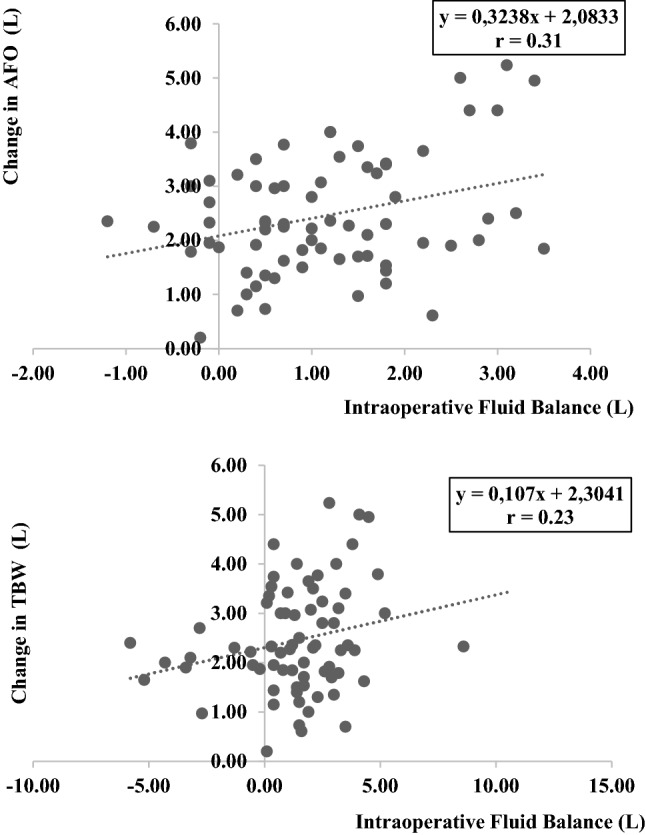
Correlation between pre- to postperative changes in BIA parameters and intraoperative fluid balance. Scatter plots. **a** Change in AFO versus intraoperative fluid balance. **b** Change in TBW versus intraoperative fluid balance. Change in BIA parameters = the difference between post- minus preoperative values. r, Pearson correlation coefficient

**Fig. 2:**
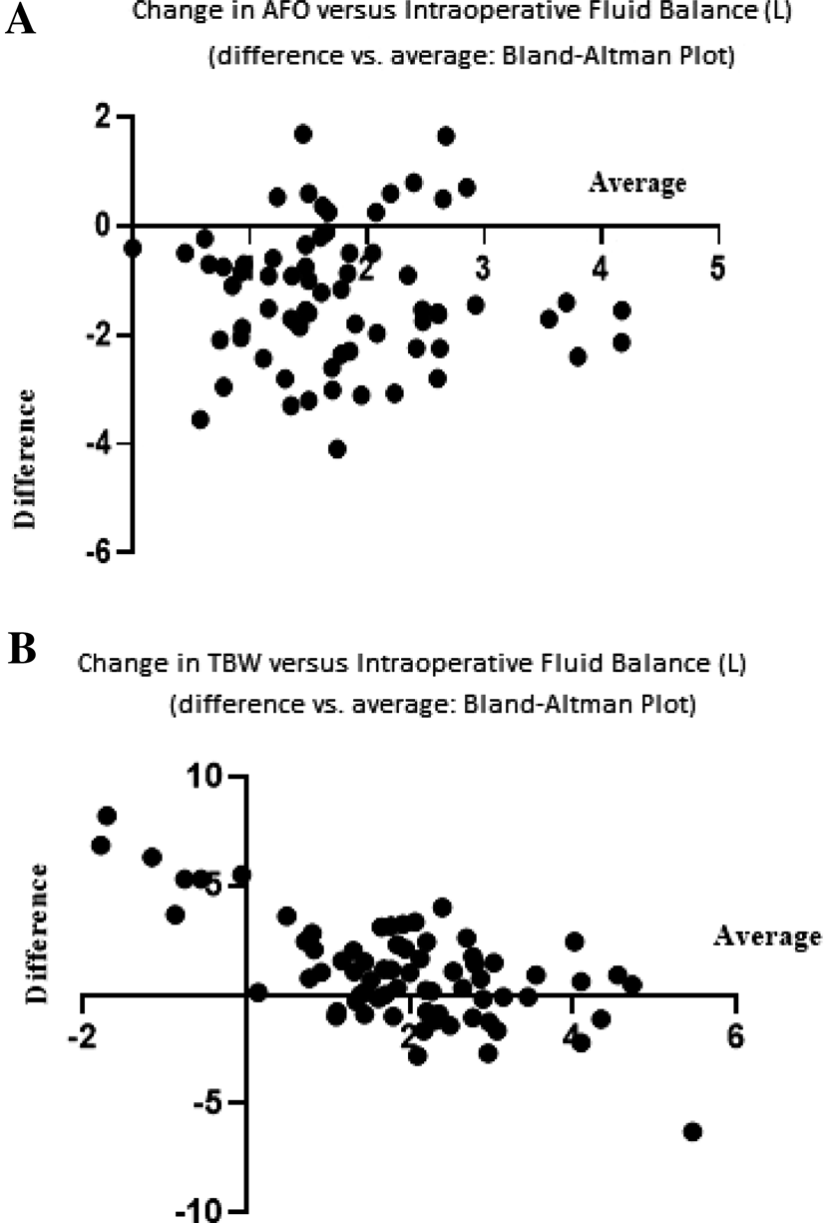
**a** Bland and Altman plot concordance between pre to postoperative changes in AFO and intraoperative fluid balance. Change in AFO = the difference between post- minus preoperative AFO values. **b** Bland and Altman plot concordance between pre to postoperative changes in TBW and intraoperative fluid balance. Change in TBW = the difference between post- minus preoperative TBW values

A significant correlation was identified between the intraoperative fluid balance and pre- to postoperative changes in TBW and AFO (r = 0.23, p = 0.04, and r = 0.31, p < 0.01, respectively) (Table 3; Figs. 1, 2). The correlation between the intraoperative fluid balance and pre- to postoperative changes in ECW did not reach statistical significance (r = 0.20, p = 0.09). Intraoperative fluid balance was non-significantly associated with pre- to postoperative changes in ICW (r = 0.12, p = 0.28) (Table 3).

**Table 3:** Correlations between pre- to postoperative changes in BIA parameters and intraoperative fluid balance

BIA parameters changes versus intraoperative fluid balance (L)	Pearson correlation coefficient (r)	p
Change in TBW, L	0.2356	**0.04**
Change in ICW, L	0.1282	0.28
Change in ECW, L	0.202	0.09
Change in AFO, L	0.3111	**< 0.01**

Change in BIA parameters = the difference between post- minus preoperative values

*AFO* absolute fluid overload, *ECW* extracellular water, *ICW* intracellular water, *TBW* total body water

#### BIA versus outcome parameters

**Fig. 3:**
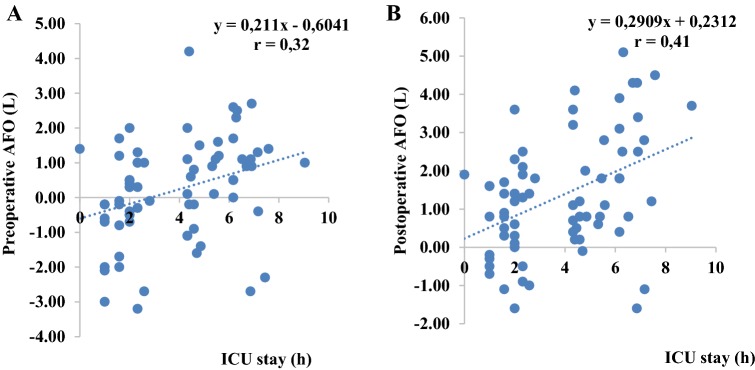
Scatter plot of preoperative AFO (**a**) and postoperative AFO (**b**) and ICU-LOS

**Fig. 4:**
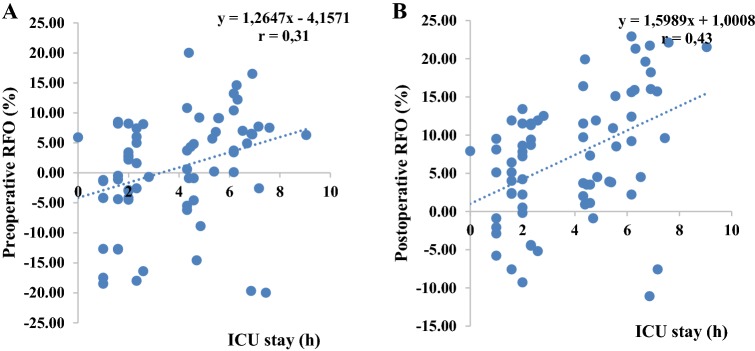
Scatter plot of preoperative RFO (**a**) and postoperative RFO (**b**) and ICU-LOS

Neither the absolute values nor the post- versus preoperative TBW, ECW ICW, AFO and RFO differences correlated with any outcome parameters. Only ICU-LOS was significantly associated with pre- and postoperative AFO (r = 0.32, p < 0.01 and r = 0.41, p < 0.001, respectively) as well as with pre- and postoperative RFO (r = 0.31, p < 0.01 and r = 0.43, p < 0.001, respectively) (Figs. 3, 4).

### Comparison between normally hydrated and fluid overloaded patients

Out of the 71 patients, 49 (69%) had a CFO < 5% (normal hydration—NH group) and 22 (31%) had CFO ≥ 5% (fluid overload—FO group). The baseline characteristics of both groups are presented in Table 1. The median surgery duration was significantly higher in the FO group (p < 0.001). During surgery, the mean fluid infusion rate was significantly different (p = 0.02): 17.5 ± 7.1 mL/kg/h in the FO group versus 14.2 ± 4.4 mL/kg/h in the NH group. During surgery, the total infusion volume was significantly higher in the FO group (5.0 ± 1.6 L) versus the NH group (3.1 ± 1.1 L) (p < 0.001), resulting in a higher positive intraoperative fluid balance: 3.4 ± 0.9 in the FO group versus 2.0 ± 0.8 in the NH group (p < 0.001). The fluid balance during the first two postoperative days was significantly different between groups: 0.9 ± 0.9 L in the FO group versus 0.1 ± 0.9 L in the NH group on day 1 (p = 0.002), and 0.5 ± 0.8 L in the FO group versus − 0.1 ± 0.6 L in the NH group on day 2 (p = 0.02). Concerning the fluid status at the end of the 2nd postoperative day, the mean CFB was significantly higher in fluid overloaded patients: 4.8 ± 1.5 L in the FO group versus 2.0 ± 1.4 L in the NH group (p < 0.001). Day 1 and 2 albumin levels were significantly lower in the FO group versus the NH group (Table 1).

Except for ICW, all other BIA parameters (TBW, ECW, ECW/ICW ratio, AFO, RFO) significantly increased postoperatively for both FO and NH groups (p < 0.001) (Table 2).

More statistically significant differences were found in the FO group versus the NH group regarding respiratory dysfunction: 11 patients (50%) from the FO group versus 3 patients (6.1%) from the NH group (p < 0.001). Last but not least, in relation to the patients’ length of stay in ICU, 13 patients (59%) from the FO group versus 6 patients (10.2%) from the NH group were required to spend two or more days there (p < 0.001).

## Discussion

To our knowledge, this is the first prospective, clinician blinded, observational study which investigates the role of bio-electrical impedance in the assessment of body fluid composition and intercompartmental fluid shifts before and after open major abdominal surgery.

Major abdominal surgery represents a traumatic insult associated with metabolic and hormonal disturbances which may ultimately induce inflammation, catabolism, and water and salt retention [36]. Volume overload in such patients is a consequence of both inadequate fluid therapy and increased capillary permeability [37]. The distinction between minor and major surgery is a pertinent one because the latter generates profound stress activation and impaired capillary permeability resulting in intercompartmental fluid shifts. Important fluid shifts is, in fact, a characteristic of major abdominal surgery, as up to 40% of patients undergoing major open abdominal surgery develop fluid overload [8, 38], which causes postoperative complications and influences mortality rates [38]. Following colon resection, a weight gain of 3–6 kg has been associated with poor outcomes [5, 18]. In the early postoperative period, patients’ bodies accumulate large fluid volumes without exhibiting clinically overt edema [8]. BIA is an objective, simple bedside technique which enabled us to see that 31% of the patients were overhydrated at the end of surgery. The cut-off BIA values used for defining normal-, over- and dehydrated patients were as for patients undergoing chronic dialysis. These values have also been used by other authors with regard to perioperative patient assessment [39].

The mean postoperative TBW and ECW increased significantly, and so did the ECW/ICW ratio from preoperative 0.85 to postoperative 0.93. This pattern is commonly seen in critically ill patients and in late stages of liver cirrhosis, heart failure, and kidney failure [30]. Further research is required to demonstrate that perioperative increases in ECW/ICW ratio could be a predictor of postoperative organ dysfunction and complications after major open abdominal surgery.

Unlike clinical examination, BIA is an objective technique that can provide numerical, credible results even in the case of patients for whom clinical assessment alone fails to identify abnormalities in fluid status. BIA has, at least theoretically, the capacity to identify both hyperhydrated and dehydrated patients. This evaluation has the advantage of early identification of hyperhydrated patients prior to the clinical occurrence of edema. BIA was validated by isotope dilution methods [40], the gold standard for assessing body fluid composition, and is commonly used to guide fluid removal in dialysis patients [41, 42].

The results of our study suggest that BIA can be used to monitor compartments volume during the perioperative period. A positive 2.4 L mean intra-operative fluid balance was detected by BIA assessment as a 1.4 L increase in TBW. The difference may be explained by insensitive losses (open peritoneum, mechanical ventilation, cutaneous losses) which were not included in the fluid balance calculation. A similar result was reached by Ernstbrunner et al. who used BIA to assess perioperative volume status in patients undergoing gynecological surgery and showed that a mean positive intraoperative fluid balance of 1.6 L resulted in a TBW increase of only 1 L and an average ECW expansion by 0.8 L [39]. At the same time, our BIA measurements indicate that the number of intraoperative fluids may result in an increase in TBW due to ECW volume expansion without a variation of ICW volume. This is consistent with the research conducted by Ernstbrunner et al. [39].

According to our analysis, intraoperative fluid balance significantly correlated with perioperative change in TBW and AFO, but did not reach statistical significance for ECW change. Using the same BIA monitor, Ernstbrunner et al. identified a stronger correlation between intraoperative fluid balance and changes in both TBW and ECW. The discrepancies between our findings and Ernstbrunner et al. can be explained by the differences between the two studies in terms of type of surgery (major open abdominal surgery versus gynecological, mainly laparoscopic procedures), patient characteristics (elderly, mostly male ASA 3 versus young ASA 1–2 females), blood loss (median blood loss 0.6 L versus significant blood loss excluded), intraoperative volume repletion strategies (crystalloids and colloids versus only crystalloids) and intraoperative insensitive losses (higher in major open abdominal surgery versus gynecological, mainly laparoscopic surgery).

Physiologically, water accounts for 50–60% of body weight and is unevenly distributed between the different compartments: intracellular water amounts to 33–40% of body weight and extracellular water represents 17–20% of body weight [43]. In our study, the preoperative mean TBW was 49%, ICW 26% and ECW 23% of body weight. These results, although different from “classical” physiology, are consistent with water composition assessed by isotope dilution, the “gold standard” technique. Chamney et al. measured the normal volumes of water compartments by isotope dilution in healthy subjects and found that, on average, TBW was 50.7%, ICW 29% and ECW 21.1% of body weight [33].

When making decisions regarding type and dose of fluid administration, one must always bear in mind that fluids are drugs nonetheless and, as such, they should be prescribed with caution considering their indications and contraindications. Given that a positive intraoperative fluid balance is common, a strategy aiming for a negative postoperative fluid balance may play an important role in preventing postoperative complications. In our study, patients in the NH group had a negative fluid balance on the 2nd postoperative day and a lower complication rate, while the FO group featured positive fluid balance and more organ dysfunctions.

At present, the accepted definition of fluid overload is a 10% increase in body weight [4, 19, 25]. On the other hand, current recommendations suggest avoiding a weight gain of more than 2.5 kg, which is a cutoff value even lower than a 5% increase in body weight for the average adult [44, 45]. Therefore, we divided our patients into two groups using a threshold CFO value of 5% in order to investigate the ability of BIA monitoring to detect fluid accumulation early.

During anaesthesia and surgery, patients often develop hypotension. Despite anaesthetics-related decreased peripheral vascular resistance and/or myocardial depression, patients often receive aggressive volume therapy. Such excessive fluid administration increases the interstitial space volume, impairing tissue oxygenation and healing, and contributing to organ dysfunction. These effects become more prominent in encapsulated organs, where small increases in interstitial volume result in significant increases in interstitial pressure and a significant reduction in capillary blood flow. Therefore, fluid overload is harmful to the kidney function, for instance [46]. Increased extravascular lung water results in impaired blood oxygenation and increased susceptibility to infection [4]. Subsequently, hyperhydration-related organ dysfunctions also have a direct impact on ICU- and hospital-LOS, as well as on mortality. Our study shows that patients with CFO ≥ 5% are at greater risk of developing respiratory dysfunction and of an increased ICU-LOS.

Despite the fact that we did not find any significant correlation between any pre- or postoperative BIA parameters and the risk of postoperative complications (AKI, respiratory dysfunctions or infections), a significant correlation was noted between the pre- and postoperative AFO, RFO and the ICU-LOS. This may be explained, on one hand, by the fact that longer ICU-LOS may be related to mild/moderate organ dysfunction, which did not reach the severity threshold of complications. On the other hand, AFO and RFO are more refined parameters of BIA measurement and may better express subclinical correlations between intraoperative water volume, distribution and mild to moderate organ dysfunction.

The impact of perioperative fluid overload on morbidity and mortality has been described in several clinical studies [3, 5–8]. Daily and cumulative assessments of fluid balance help detect fluid overload. In our study, we found that higher CFO associated with an increased risk of respiratory dysfunction and a longer stay in ICU. However, the accuracy and reliability of fluid balance recordings in postoperative patients have already been questioned [47]. While the amount of given fluids is well known, the output may be difficult to accurately record mainly due to insensible losses, which are only estimated and, therefore, frequently overlooked. Our study shows that, as a readily available, bedside diagnostic tool, BIA may be a promising way to monitor the patient’s hydration status. The single postoperative BIA assessment, as one limitation of our study, may explain the lack of significant correlations with postoperative outcomes. Extended BIA monitoring during the postoperative period might yield additional data and facilitate assessment of the prognostic value of this approach.

BIA is a non-invasive, inexpensive technique with limited inter-observer variation. It performs well for both healthy subjects and chronic patients with major but subtle water distribution disturbances [48]. In such cases as the latter, the calculations result in reproducible and reliable measurements. BIA is considered unreliable in patients with highly altered hydration state, for which interpersonal and intrapersonal fluid status variations are high and subject to change, sometimes over short time periods. At present, BIA can only be considered as a research tool for studying such patients [30, 49].

The accuracy of BCM measurement may be altered by electrical interference; therefore, BCM is not recommended with ICU patients. Dewitte et al. showed that the BCM device is safe and feasible with ICU patients, while data reproducibility was very good, whether with or without ICU monitoring and mechanical ventilation [50].

## Limitations

Our study is subject to a number of limitations. Firstly, since ours is a single-center study with a relatively small number of patients, our findings need to be corroborated with those by other, larger or multi-centric studies. Secondly, the study was a non-interventional, observational pilot study, and as such, no cause-effect relationship can be assumed. Also, the patents were not classified according to the accepted definition of fluid overload—a 10% increase in body weight—and we used a 5% threshold in order to investigate if BIA monitoring may help with the early detection of fluid accumulation. Moreover, this study included patients with open major abdominal surgery, of whom only 26.7% had an ICU stay ≥ 2 days. Finally, the postoperative BIA measurements were performed only once during the first hour. Extending BIA fluid monitoring in the postoperative period might have allowed for a more in-depth assessment of fluid compartment status and shifts, as well as correlation with patient outcomes.

## Conclusion

In conclusion, BIA is a simple, non-invasive, low-cost bedside technique which may prove for the perioperative assessment of volume status in open major abdominal surgery. For instance, fluid overloaded patients are more likely to require longer ICU hospitalization, and BIA may be used to identify such situations. Further studies are required to evaluate whether BIA-guided perioperative fluid administration may improve the postoperative volume status and clinical outcomes in patients undergoing major open abdominal surgery.
